# CoQ10 and Aging

**DOI:** 10.3390/biology8020028

**Published:** 2019-05-11

**Authors:** Isabella Peixoto de Barcelos, Richard H. Haas

**Affiliations:** 1 Department of Neurosciences, University of California San Diego, San Diego, CA 92093-0935, USA; ibarcelos@ucsd.edu; 2 Department of Pediatrics, University of California San Diego, San Diego, CA 92093-0935, USA

**Keywords:** coenzyme Q10, aging, age-related diseases, mitochondrial dysfunction

## Abstract

The aging process includes impairment in mitochondrial function, a reduction in anti-oxidant activity, and an increase in oxidative stress, marked by an increase in reactive oxygen species (ROS) production. Oxidative damage to macromolecules including DNA and electron transport proteins likely increases ROS production resulting in further damage. This oxidative theory of cell aging is supported by the fact that diseases associated with the aging process are marked by increased oxidative stress. Coenzyme Q10 (CoQ_10_) levels fall with aging in the human but this is not seen in all species or all tissues. It is unknown whether lower CoQ_10_ levels have a part to play in aging and disease or whether it is an inconsequential cellular response to aging. Despite the current lay public interest in supplementing with CoQ_10_, there is currently not enough evidence to recommend CoQ_10_ supplementation as an anti-aging anti-oxidant therapy.

## 1. Introduction

CoQ_10_ was first described in 1955, named ubiquitous quinone, a small lipophilic molecule located widely in cell membranes [1], and in 1957 its function as an electron carrier in the mitochondrial electron transport chain was reported [2]. The role in human disease was unknown for 20 years until in 1986 a benefit of CoQ_10_ treatment was reported in Kearns–Sayre syndrome [3]. Initially, therapeutic use of CoQ_10_ was focused on the oxidative phosphorylation (OXPHOS) defects in which there is documented CoQ_10_ deficiency [4] and in the group of CoQ_10_ synthesis disorders [5]. These conditions provided evidence for efficacy and safety of treatment with CoQ_10_ [6]. Subsequent larger-scale trials in Parkinson disease [7] and other neurodegenerative diseases have shown safety but no convincing benefit.

In the last decade, CoQ_10_ functions in membranes throughout the cell where antioxidant and signaling roles predominate have been of increasing interest [8]. There is growing evidence that oxidative stress is a major component of cellular senescence [9]. This multifactorial process involves DNA injury [10], protein and lipid damage, and activation of signaling pathways associated with aging [11]. Recently, the CoQ_10_ antioxidant effect has been shown to reduce markers for cardiovascular disease (CVD) and inflammation, the main components of atherosclerotic vascular disease [12].

It is suggested that CoQ_10_ supplementation can improve the symptoms of mitochondrial diseases and of aging because of an improvement in bioenergetics [12,13].

Our objective is to review, from a translational perspective, data regarding the association of CoQ_10_ and aging. Are the aging process and mitochondrial progressive failure related and can CoQ_10_ supplementation decelerate aging?

## 2. CoQ_10_

### 2.1. What Is It?

Coenzyme Q10, CoQ_10_ or ubiquinone (2,3 dimethoxy-5-methyl-6-decaprenyl-1,4-benzoquinone) is a small lipophilic structure, composed of a benzoquinone ring and an isoprenoid side-chain and it is found universally in cell membranes. In humans, synthesis occurs utilizing a collection of enzymes (complex Q) located in the mitochondrial matrix membrane [14]. The benzoquinone ring is derived from 4-hydroxybenzoic acid, while 10 isoprenes are derived from mevalonic acid (from the cholesterol synthesis pathway). The quinone ring is the functional group in the molecule, responsible for carrying electrons to complex III. CoQ_10_ (ubiquinone) is reversely reduced to ubiquinol. The polyisoprenoid tail is very lipophilic and localizes to hydrophobic membranes. The length of the isoprenyl chain is variable between species with 10 isoprenes forming human CoQ_10_ whilst rodents predominantly have CoQ_9_ [15].

### 2.2. Function

CoQ_10_ is widely distributed in all cell membranes and forms a critical component of the electron transport chain (ETC) transporting electrons between complexes I/II and III [13]. In rat liver the largest ubiquinone (CoQ_9_) concentration is found in the Golgi vesicles (2.62 μg/mg) followed by mitochondrial matrix membrane and lysosomes (with levels of 1.86 in each structure) [16].

**Figure 1:**
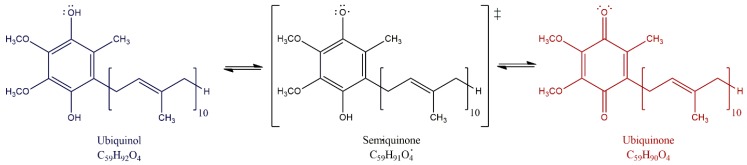
Redox forms of CoQ_10._ ubiquinone (oxidized form), ubiquinol (reduced form), and semiquinone (semi-oxidized). The Q cycle within the matrix membrane allows proton transfer from the mitochondrial matrix to the intermembrane space helping to generate the electrochemical gradient for ATP production.

The major function of ubiquinone is in the mitochondrial ETC. CoQ_10_ accepts electrons from different donors, including complex I (reduced nicotinamide adenine dinucleotide [NADH]-coenzyme Q oxidoreductase), complex II (succinate dehydrogenase), the oxidation of fatty acids and branched-chain amino acids via flavin-linked dehydrogenases and electron transfer factor Q oxidoreductase (ETF-QO) to complex III (ubiquinone-cytochrome c oxidoreductase) [17,18]. CoQ_10_ cycles between its three chemical forms: completely oxidized (ubiquinone), a semi-oxidized intermediate free radical (semiquinone) and a completely reduced form (ubiquinol) as shown in Figure 1 [19,20]. By moving within the mitochondrial membrane, the proton-motive Q cycle allows proton pumping at complex III helping to generate the proton motive force for adenosine triphosphate (ATP) production.

The interaction of the CoQ pool with the ETC has in recent years been shown to be more complex with the recognition that mitochondrial supercomplexes composed mostly of complexes I/III, I/III/IV, and III/IV interact physically forming respirasomes. It seems likely that both single complexes within the matrix membrane and supercomplexes coexist in a dynamic state. The factors controlling assembly and disassembly of supercomplexes are not known although cardiolipin appears to play a role. There is evidence that in the I/III supercomplex intercomplex binding of CoQ shuttles electrons from complex I to III. This bound CoQ may be in equilibrium with the free CoQ pool. [21].

CoQ10 supplementation has been shown to have epigenetic effects in genes involved with signaling, intermediary metabolism, transport, transcription control, disease mutation, phosphorylation, and embryonal development indicating a role in modulation of gene expression [22,23].

**Figure 2:**
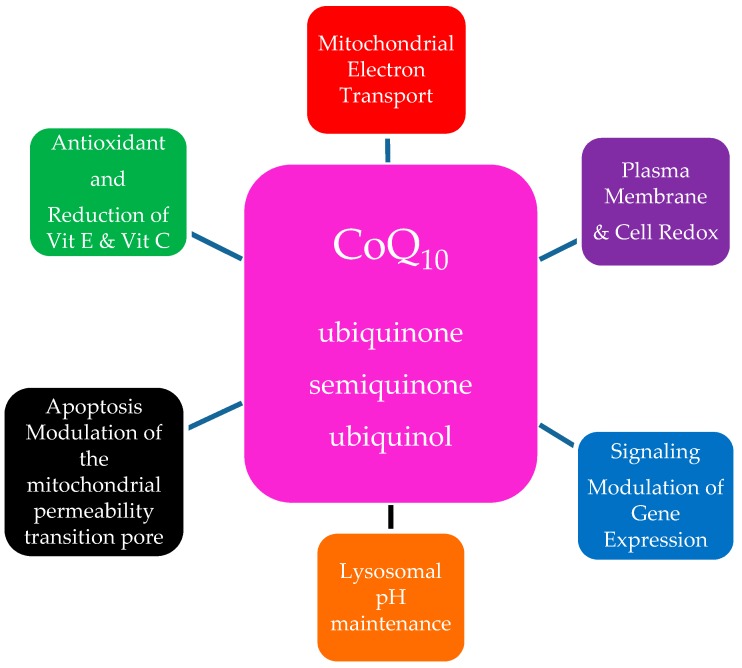
The Multiple Roles of Ubiquinone in the Cell.

In addition to its major function in the ETC, CoQ_10_ has an important anti-oxidant role stabilizing the plasma membrane and other intracellular membranes protecting membrane phospholipids from peroxidation [13]. Ubiquinone and semiquinone are also involved with recycling of other anti-oxidant molecules, reducing α-tocopherol and ascorbate contributing to redox balance in the cell. Diminished CoQ_10_ levels in aging likely contribute to membrane peroxidation injury. There is evidence that part of its anti-oxidant effect occurs by enhancing the enzymatic activity of the antioxidant proteins superoxide dismutase and glutathione peroxidase [24]. Recent publications associate ubiquinol with protection of plasma low density lipoproteins (LDL) from oxidation, an important anti-atherogenic effect [25]. The pro-oxidant role of CoQ_10_ is a signaling function involved in gene expression but the mechanism of this function is not fully understood [26,27]. Other functions include modulation of the permeability transition pore, thus playing a role in apoptosis [28]. CoQ_10′_s main functions are summarized in Figure 2.

Chronic inflammation is a frequent aging-related problem. CoQ_10_, by reduction of free radicals, reduces the activation of NF-κB (nuclear factor kappa-light-chain-enhancer of activated B cells) cells and consequently reduces the release of pro-inflammatory cytokines mainly tumor necrosis factor alpha (TNF-α) and interleukin 6 (IL-6) [29]. Aging-related reduced CoQ_10_ levels may contribute to inflammation and there is accumulating evidence of secondary anti-inflammatory effects of CoQ_10_ supplementation. A recent meta-analysis provided evidence that CoQ_10_ supplementation significantly reduced the inflammatory markers CRP (C-reactive protein), IL-6 and TNF-α [30]. Another recent publication reported that patients with metabolic diseases (obesity, type 2 diabetes, metabolic syndrome, cardiovascular disease, and nonalcoholic fatty liver disease) had a significant decrease in TNF-α plasma levels with CoQ_10_ supplementation but not CRP or IL-6 [31]. CoQ_10_ was found to have an anti-inflammatory function via epigenetic effects on expression of genes related to NFkappaB1 (NFk-B1) [32]. CoQ_10_ has a hepatoprotective and neuroprotective effect in a rat model of non-alcoholic steatohepatitis [33] apparently through an adenosine 5′ monophosphate-activated protein kinase (AMPK) activation mechanism and in humans a randomized trial showed that supplementation of CoQ_10_ improved biomarkers for inflammation in nonalcoholic fatty liver disease (NAFLD) [34]. CoQ_10_ supplementation in patients with antiphospholipid syndrome has been found to attenuate levels of pro-inflammatory and thrombotic markers, with evidence of endothelial and mitochondrial function improvement [35]. In Down syndrome patients, chronic neuro-inflammatory changes have been proposed as a possible accelerator of Alzheimer disease [36]. These include high levels of interleukin 6 and tumor necrosis factor α along with decreased levels of CoQ_10_. A positive correlation between CoQ_10_ and intelligence quotient levels was also reported [37]. CoQ_10_ treatment in Down syndrome cells is associated with improved DNA repair mechanisms and DNA protection [38].

Cardiovascular disease is a common aging-related problem. There is a considerable body of evidence supporting a role for CoQ_10_ in cardiovascular function including a correlation of low endomyocardial levels with severity of heart failure and an improvement in cardiac contractility with CoQ_10_ treatment [39]. Improvement in lipid profiles (a major contributor to cardiovascular disease) has been reported with CoQ_10_ treatment [40].

CoQ_10_ has other important functions, participating in metabolic pathways as an electron receptor: (1) CoQ_10_ is a co-factor for dihydro-orotate dehydrogenase, an enzyme involved in the de novo pyrimidine biosynthesis [41]. (2) During the process of sulfide oxidation CoQ_10_ accepts electrons from the enzyme sulfide-quinone reductase to convert sulfide into thiosulfate [42]. (3) The oxidation from choline to glycine is catalyzed by choline dehydrogenase in the inner mitochondrial membrane, and CoQ_10_ is proposed to be the electron acceptor for this reaction [43]. (4) Proline dehydrogenase donates electrons from FAD and NAD+ to CoQ_10_ during process of synthesis of proline and arginine [21,44].

### 2.3. Sources of CoQ_10_

#### 2.3.1. Internal Biosynthesis 

CoQ_10_ is the only lipid-soluble antioxidant synthetized by the human body [13]. The majority of CoQ_10_ comes from the internal synthesis, from tyrosine or phenylalanine (benzoquinone ring) and mevalonic acid (isoprenoid side-chain). Synthesis occurs in all tissues studied. In humans synthesis occurs utilizing a collection of enzymes (complex Q) located in the mitochondrial matrix membrane and in the endoplasmic reticulum The mevalonic acid pathway is responsible for cholesterol synthesis with 3-hydroxy-3-methyl-glutaryl-coenzyme A (HMG-CoA) reductase (the site of statin inhibition) as the regulatory step. CoQ_10_ derived from dietary intake becomes more important with aging as endogenous production decreases [13].

**Figure 3:**
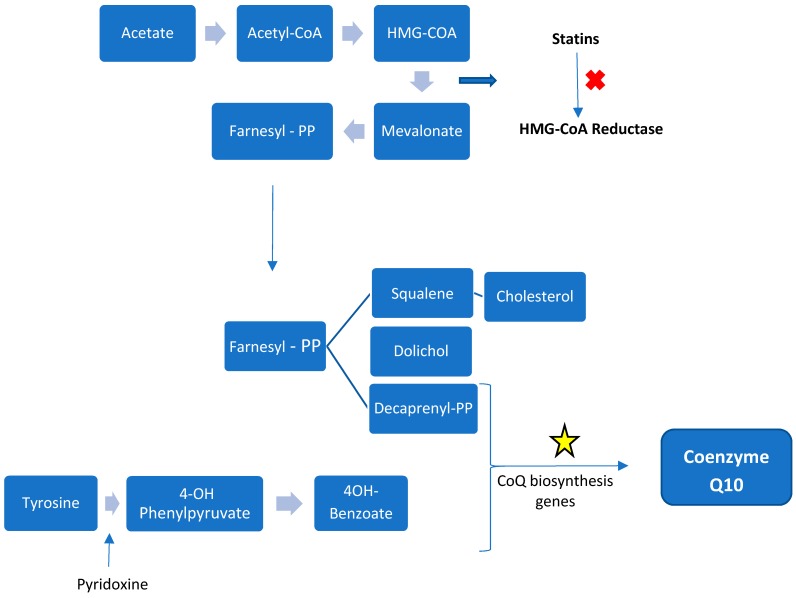
Schematic representation of CoQ biosynthesis. The isoprenoid side-chain derives from the cholesterol and dolichol synthetic pathway from mevalonate, the benzoquinone ring is derived from tyrosine metabolism. The star symbolizes the genes involved in the final synthesis of CoQ_10_.

At least 14 genes are involved in CoQ_10′_s biosynthesis in yeast with 18 genes so far reported in humans. Many are homologues of genes identified in c. cerevisiae. These synthetic proteins are nuclear encoded and require mitochondrial targeting sequences for entry into the matrix or the inner mitochondrial membrane [14]. There is assembly of many of the components into a ‘supercomplex’ termed Complex Q in the mammal. Mitochondrial synthesis is thought to occur in all cells containing mitochondria but also in other organelles including the endoplasmic reticulum and peroxisomes [45]. Figure 3 summarizes the mammalian CoQ_10_ biosynthesis pathway.

#### 2.3.2. External Sources

CoQ_10_ is widely found in many animal protein sources (pork, lamb, beef, chicken, fish), vegetables (spinach, pea, broccoli, cauliflower), fruits (orange, strawberry, apple) and cereals (rye, wheat) [13]. Heart, chicken leg, herring, and trout contain particularly high amounts of CoQ_10_. Daily intake between 3 and 5 mg is considered adequate and whilst external supplementation increases plasma levels, supplementation was not thought to increase tissue levels of CoQ_10_ in tissues with normal synthetic capacity [46], although evidence of treatment efficacy in a variety of human diseases does suggest that tissue uptake can occur [13,47].

#### 2.3.3. Absorption and Transport

CoQ_10_ absorption is slow and occurs in the small intestine; in its reduced form, ubiquinol is 3 to 4 times better absorbed than the oxidized form, ubiquinone [48]. The absorption of CoQ_10_ can be increased if administered with food intake, mainly with lipids because of its lipophilic structure [13,49]. After absorption by the enterocytes, CoQ_10_ passes through lymphatic vessels and reaches the plasma, where it circulates bound to lipoproteins (LDL). Because of this, plasma measurements of CoQ_10_ should be corrected for lipoproteins levels. Between 80 and 95% of plasma circulating CoQ_10_ is in the reduced ubiquinol form. [48,50].

### 2.4. Tissue Levels and Distribution of CoQ_10_

Although the major CoQ_10_ plasma form is ubiquinol, laboratory measurements, in general, report the CoQ_10_ total level [50]. Lymphocyte and platelet levels may give some insight into levels in less accessible tissues such as heart, muscle, and brain [51]. The CoQ_10_ level varies in different tissues. Tissues with a higher metabolic rate and mitochondrial content (heart, kidney, liver, and muscle) have high levels of CoQ_10_, for example the level is 8 μg/g in lung and 114 μg/g in heart [13]. Tissue levels largely reflect the results of synthesis and degradation of CoQ_10_. Following intestinal absorption, liver and lipoprotein concentrations increase, but without a change in the level in heart and kidney noted in early studies [52]. However, there is some evidence that chronic administration can increase tissue levels [53]. In a study where rats were chronically fed a large dose of 150 mg/kg/day of CoQ_10_ for 13 weeks, small but significant increases in both CoQ_9_ and CoQ_10_ were found in all tissues measured [54]. No such data exist for young or aged human tissues; however, the accumulated evidence of benefit of CoQ_10_ therapy in human disease states does suggest that tissue levels can be increased by oral administration. The number, size, and structure of the mitochondria in each cell determines the tissue level of CoQ_10_ with highest levels found in tissues with high energy demands and a high mitochondrial content [13,48].

The gold standard for the diagnosis of CoQ_10_ deficiency is based on the measurement of muscle level by high-performance liquid chromatography (HLPC) [55,56]. Plasma levels of CoQ_10_ range between 0.40 and 1.91μmol/L (0.34–1.65μg/mL) in controls [57] but do not match tissue levels, reflecting consumption much more than endogenous synthesis. The diagnosis of CoQ_10_ deficiency requires tissue measurement [58]. There is evidence suggesting that the CoQ_10_ level in mononuclear cells can be correlated to muscle measurements [45,56]. Plasma CoQ_10_ levels are increased in some physiological conditions (cold adaptation and exercise) and in some diseases (paraneoplastic nodules, Alzheimer’s disease, and prion disease). Levels are decreased in aging. [45].

### 2.5. Causes of Reduction

Conditions associated with CoQ_10_ deficiency can be divided into three main groups: (1) CoQ_10_ nutritional deficiency, including intake of CoQ_10_ itself and nutrients and vitamins necessary for its synthesis (vitamin B6 is a cofactor in the pathway of CoQ_10_ biosynthesis); (2) CoQ_10_ synthesis genes (*COQ* family genes: *COQ1* and the Complex Q genes including the mammalian homolog of the yeast Coq11 gene; the Complex I subunit *NDUFA9*), and acquired disorders impairing CoQ_10_ synthesis (statin use) [59,60,61]; and (3) medical conditions associated with decreased levels of CoQ_10_ [45,46]. In this last category, a variety of conditions have been reported with low CoQ_10_ including neurodegenerative disorders Friedreich’s ataxia, Nieman–Pick type C disease, and Parkinson Disease. In other disorders such as Alzheimer disease, diabetes, cancer, fibromyalgia, and cardiovascular diseases, elevations in plasma CoQ_10_ levels may be a stress response. [45,46]. Defects in genes which may be associated with reduced CoQ_10_ levels can be included in this group (*APTX*, *ETFDH*, *BRAF*).

Special attention should be given to the important observation that CoQ_10_ deficiency is potentially reversible if the supplementation starts before the appearance of the symptoms, when brain and kidney have not sustained permanent damage [46,62].

### 2.6. Supplementation

#### 2.6.1. Dosing

A recent trend over the last few years has been to supplement adult patients with mitochondrial diseases with high doses of oral CoQ_10_ or ubiquinol, up to 1200 mg/day or higher. For the pediatric population, doses between 5 and 10 mg/kg/day of ubiquinol are recommended [63]. For a dose of 10 mg/kg/day, plasma levels range between 5 and 10 µg/mL 3–4 weeks after the beginning of the supplementation [53,63,64]. As explained above, there are important differences in bioavailability of the different formulations of CoQ_10_ used; ubiquinol (the reduced form) is 3 to 4 times better absorbed than ubiquinone (oxidized form) [48]. Primary CoQ_10_ diseases tend to respond to supplementation but may require very high doses. Also, some patients with secondary CoQ_10_ dysfunction were reported to improve some symptoms with CoQ_10_ supplementation. Examples are cardiomyopathy in organic acidurias (25 mg/kg/day with improvement in the cardiomyopathy) [65], glutaric acidemia type II (500 mg per day of CoQ_10_ along with riboflavin with improvement in strength, lactate, and creatine kinase levels) [59], ataxia oculomotor apraxia type 1 (200–600 mg with reported improvement in strength, ataxia, and cessation of seizures in one patient) [60], GLUT-1 deficiency (30 mg/kg/day with improvement in the ataxia and nystagmus) [66].

Exogenous administration of CoQ_10_ reportedly does not raise tissue levels above normal in healthy young individuals, except for two tissues (liver and spleen) [13]; however, this traditional view may be wrong given the improvement in multi-organ symptoms in a variety of disorders with mitochondrial dysfunction when treated with CoQ [67].

#### 2.6.2. Safety and Adverse Events 

Although the majority of studies have not shown convincing enough scientific evidence to support treatment with CoQ_10_ in specific diseases, they do provide evidence that oral supplementation is safe and well tolerated. One of the largest trials was a phase III randomized, placebo-controlled, double-blind clinical trial at 67 North American sites by the Parkinson Study Group using doses of CoQ_10_ up to 2400 mg/day demonstrated the safety of this dose [7]. Evidence suggests that supplementation does not inhibit endogenous production [13]. Previous studies had reported only mild side effects such as gastrointestinal symptoms, mainly nausea, with CoQ_10_ supplementation [13,68].

## 3. Aging

### 3.1. Physiology of Mitochondrial Involvement in the Process of Aging

Human aging is a normal multifactorial process resulting from the interaction of genetic and environmental factors. It is characterized by multi-organ system functional decline in association with the risk of age-related diseases (dementia, neurodegenerative disorders, osteoporosis, arthritis, diabetes, cardiovascular disease, age-related hearing loss, and cancer) [13,68,69,70,71].

A common hypothesis to explain some of the pathophysiology of age and degenerative diseases is an oxidative imbalance between the production of reactive oxygen species (hydrogen peroxide: H2O2, the oxygen-derived free radicals superoxide: O2•−, and hydroxyl radical: HO•), and antioxidant mechanisms such as superoxide dismutase, catalase, glutathione peroxidase, ascorbic acid, tocopherol, glutathione, and CoQ_10_, leading to a state of oxidative stress [72,73,74,75,76,77,78,79,80]. A number of animal models support this theory with shortened survival in mice lacking superoxide dismutase 1 (SOD1) and a lethal phenotype in mice lacking superoxide dismutase 2 (SOD2) [81,82].

As mitochondria are the main source of reactive oxygen species (ROS) production though OXPHOS supercomplex activity in the cristae of the inner mitochondrial membrane (mainly at complex I and III), this organelle is the major target of ROS damage. Mitochondrial DNA (mtDNA) is particularly vulnerable with a high mutation rate and limited mtDNA repair mechanisms [69,74]. The continuous production and accumulation of mitochondrial ROS is the basis for “the free radical theory of aging” [70,71]. Although the accumulation of ROS has a major effect on DNA (strand breaks, oxidation of bases, damage in sites coding for ETC proteins), other structures of the cell are also damaged: lipids, membranes, proteins (leading to dysfunction of the ETC, inadequate ATP production, and further ROS production) [72,73,74,75]. There is evidence that impaired mitochondrial machinery produces more oxidative stress and more ROS production, resulting in a vicious cycle [76,83]. Electrons leaking from impaired OXPHOS react with oxygen molecules to form the free radical superoxide [80]. There is also an effect on mitochondrial dynamics with impairment of fission, contributing to mitochondrial enlargement, which reduces recycling through mitophagy leading to a reduction in ATP generation [73]. Impairment of the mitochondrial–lysosomal axis occurs with aging, with accumulation of lipofuscin inside lysosomes with senescence. Lipofuscin accumulation is postulated to limit the ability of lysosomes to participate in mitophagy [73].

Considerable evidence supports the relationship between ROS accumulation and mitochondrial dysfunction leading to aging (the mitochondrial free radical theory of aging): ROS production increases in aged humans and animals [70,84], imbalance in the levels of pro and anti-oxidant substances occurs [85] with high levels of oxidized and damaged macromolecules (proteins, lipids, and DNA) [86]. There seems to be a convincing relationship between high ROS levels and longevity in humans and animals [87]; however, the exact role of ROS remains unclear as some animal models report a failure to increase longevity with ROS reduction and others link high levels of ROS and longevity. It remains unclear what the contribution of ROS generation is versus epigenetic factors modulating genes related to the protection from effects of aging [77,88,89]. Evidence against the oxidative theory of aging comes from some animal models where longevity is unaffected by increased ROS production. Some of these studies are detailed below.

Growing evidence supports the idea that increased levels of ROS are associated with the specific biochemical pathway that improves longevity, at least in some species [90]. *C. elegans*, a nematode mutant model clk-1 (*COQ7* equivalent gene in humans), has higher longevity associated with higher ROS production. *C. elegans* with a point mutation in the gene isp-1, responsible for an iron-sulfur mitochondrial complex, also demonstrate an increase in life span [91]. These observations point out the multiple roles of ROS particularly as signaling molecules triggering protective pathways. Some mouse models of defective CoQ synthesis are difficult to square with the oxidative theory of aging. Mice with only one copy of the gene *Mclk1* (equivalent of mammals *COQ7* and *C. elegans* clk-1 model) have higher production of ROS in the mitochondria (although a normal level of total ROS in the body), a higher level of protection of the immune system (from some infections and also tumorigenesis) and increased longevity [92]. Homozygous knockout of the *Mclk1* gene is embryonic lethal but utilizing a Tamoxifen dependent transgene mouse KO activated at 2 months of age a multisystemic disorder (heart, kidneys, and skeletal muscles) with a decline in ubiquinol levels is produced. At 8 months this “ubiquinol deficit” animal presented normal levels of some factors associated with oxidative stress (catalase, F_2_-isoprostanes, DNA oxidative damage, SOD1, and SOD2). Diet supplementation at 9 months with an analogue of the ubiquinol precursor 4-hydroxybenzoic acid rescued the clinical phenotype [93]. Another mouse model heterozygous for the Sod2 gene (with decreased Mn-superoxide dismutase activity) does show oxidative injury (increased tumor incidence and DNA damage) but does not decrease longevity [94]. The concept that mitochondrial dysfunction may be a consequence of aging factors rather than a cause is also supported by observations of sarcopenia in rat and human muscles with other factors such as denervation playing a role [95].

Further studies are needed to understand the balance of ROS as an agent of oxidative injury and its signaling epigenetic role modulating genes related to the protection of effects of aging. It may be that after the saturation of the mechanisms of protection, the ROS-stress protective cascade can no longer prevent oxidative damage [88].

It is not surprising that given the contradictory evidence for a central role for ROS in aging, evidence supporting the utility of anti-oxidant therapies in aging (such as CoQ_10_) remains unclear.

### 3.2. CoQ_10_ and Aging, CoQ_10_ Deficiency in Advanced Age, Evidence for Beneficial Supplementation 

Published results from various research groups about CoQ levels and lifespan are often at variance, model dependent and do not support a similar pattern in all species [96,97,98].

#### 3.2.1. *C. elegans*

Studies in the nematode *C. elegans* have produced unexpected results compared to mammals. As discussed above, a nematode mutant model clk-1 (mammals *COQ7* equivalent gene), with low production of CoQ_8_, has an extension in longevity compared with the wild strain but requires dietary Q supplementation [99]. This diet does trigger a Dauer long-lived larval anerobic state. Other studies with CoQ gene knockouts confirm that deficiency in CoQ (less than 50%) also leads to an increase in lifespan, and a possible explanation is that less ROS production occurs in the case of moderate CoQ deficiency [100,101], but with more severe depletion of CoQ, longevity would be affected [100,102]. This finding was also confirmed in human cells, with two different studies from the same group reporting that fibroblasts with mutations on *PDSS2* (homologue of yeast coq1) with less than 12 and 20% of CoQ_10_ of control cells had decreased synthesis of ATP without increase in the levels of ROS. However, when the defect was of 30%, with partial defect in the synthesis of ATP the levels of ROS were higher. The explanation proposed centered on the severity of the deficiency of coenzyme Q as an oxphos modulator [103,104].

#### 3.2.2. Rodent Models

**Figure 4:**
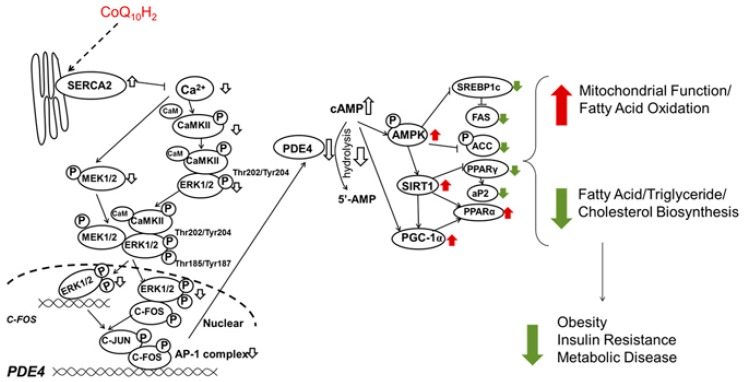
Proposed mechanism by which ubiquinol improves metabolic function and inhibits insulin resistance in KKAy mice (a mouse model of obesity and diabetes). Ubiquinol inhibited phosphorylation of CaMKII (Ca2+/calmodulin-dependent protein kinase II) in the liver resulting in inhibition of C-FOS transcriptional activity and inhibition of PDE4 gene expression. Increased cAMP increases AMPK (AMP-activated protein kinase) activity resulting in SIRT1 and PGC-1α increased mitochondrial function and inhibition of lipid synthesis. (Adapted from Xu H. et al. 2017 [106] with permission).

A diet supplemented with Ubiquinol-10 in the senescence-accelerated mouse prone 1 (SAMP1) reduced markers of oxidative stress (ratio of reduced and oxidized glutathione—GSH/GSSG), decelerated the normal decline in expression of genes (*Sirt1, Sirt3,* and *Pgc-1a,* and *Ppara*), and their respective proteins related to mitochondrial function during aging [105]. This treatment also increased auditory brainstem response hearing loss. The proposed mechanism of CoQ_10_ benefit as an anti-oxidant agent in this aging mouse is through cyclic adenosine monophosphate (cAMP). There is an enhancement in sirtuin genes, and PGC-1α (peroxisome proliferator-activated receptor gamma coactivator 1-alpha) with increased complex I and IV activity and reduce oxidative stress. Despite all these beneficial effects, there was no significant change in the overall lifespans compared to the control animals [105]. These findings of increased cAMP, SIRT1 (sirtuin 1) expression, and PGC-1α in reducing parameters of oxidative stress and increasing mitochondrial function were also confirmed in other experiments with ubiquinol supplementation in senescence-accelerated mice, with added benefits in obesity, insulin resistance, and metabolic syndrome, (hypothesized mechanism in Figure 4 [106]). PPARα (peroxisome proliferator-activated receptors) signaling and lipid metabolism gene expression changes in liver of C57BL6J mice was reported after one-week supplementation with ubiquinol (250 mg/kg BW/day) [23].

Interestingly mice with a single copy of the Coq7 synthesis gene demonstrated increased longevity [107]. However the mechanism is unclear and several studies report that dietary supplementation with CoQ_10_ or ubiquinol in various rat models improves mechanisms involved with mitochondrial biogenesis, including parameters of oxidative stress [105,108,109,110,111].

#### 3.2.3. Mammals and Tissue CoQ levels

In mammals there is a tendency to ubiquinone to be reduced with age, but this finding depends on the tissue investigated and also the species [97]. Early studies suggested that tissue levels of ubiquinol were endogenously produced with little change with dietary supplementation except in liver and spleen; however, subsequent studies in rodents confirm that oral supplementation with CoQ_10_ does increase tissue levels of both CoQ_10_ and CoQ_9_ in skeletal muscle, heart, and kidney [26], and when high doses (200 mg/kg) are used for 2 months in rats, brain levels are increased and are neuroprotective [112].

In humans, there is also a lack of consistent data. One study did not find a relationship between aging and CoQ_10_ plasma levels in elderly women [113]. Others report that plasma and tissue levels change over time, with a peak in pancreas and adrenal by 1 year of age and in the brain, heart, and lung by 20 years. After this peak, levels decrease over time [114]. A decrease in brain CoQ_10_ was confirmed in other studies [115,116]. Only 50% of the myocardial CoQ_10_ endogenous production remains by the age of 80 [114]. Serum total CoQ_10_ and ascorbic acid levels were decreased in centenarians compared with 76-year-old controls. An elevation of the CoQ_10_ binding protein prosaposin was also noted presumably in an attempt to compensate for low CoQ_10_ levels [117]. The authors conclude that CoQ_10_ supplementation could be beneficial for centenarians. However, it is not known if low tissue and plasma CoQ_10_ levels contribute to or are a side effect of aging.

**Figure 5:**
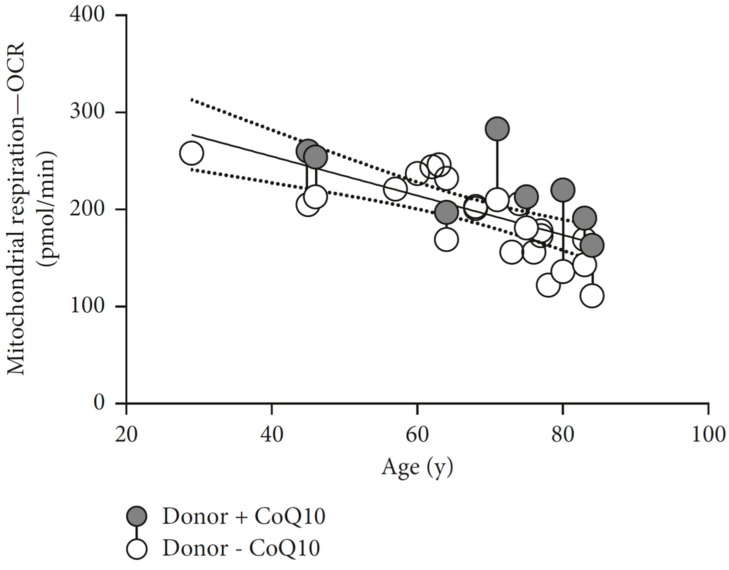
Age-related decline in the oxygen consumption rate in epithelial tissue measured 16 h after collection in a Seahorse XF analyzer. The gray circles show treated samples, the white circles untreated samples, and the connected circles represent samples from the same donor. Significant improvement with 100 μM CoQ_10_ incubation. (Adapted from Schniertshauer et al. 2018 [70] with permission).

Reduction of CoQ_10_ with age is postulated to result from reduction in biosynthesis coupled with an increase in degradation attributed to age-related modification in lipid membranes, which alters quinone behavior [118]. This reduction of CoQ_10_ levels has a tissue/organ specificity with reported high levels of CoQ_10_ in the brain mitochondria from old rats [26], and reduced level in the muscle [98]. A recent paper showed age-related reduction in mitochondrial respiration parameters and ATP production in epithelial cells, rescued with CoQ_10_ administration. Both cited that parameters decrease as age increases, as seen in Figure 5, with the estimate of 10% reduction in mitochondrial respiration every ten years [70].

High CoQ_10_ concentration was reported to be associated with higher physical activity, lipid peroxidation, and lower oxidized LDL levels in elderly people. The same publication describes higher levels of plasma CoQ_10_ in elderly (more than 50 years old) people compared with young (less than 30 years) [119]. The same group confirmed lower lipid peroxidation and lower oxidized LDL in young adults and also showed that CoQ_10_ is lower in obese elderly patients [120].

In another study, prolonged CoQ_10_ supplementation for 4 years in community-dwelling elderly was associated not only with an improvement in health-related quality of life but more importantly with a lower “more days out of hospital“ rate. In this study, subjects were co-supplemented with selenium [121].

Although the association between CoQ_10_ and muscle power is not well established, one study showed a positive relation between CoQ_10_/cholesterol levels and hand grip, and lower ubiquinol levels in patients with less muscle strength. This study was undertaken to evaluate a possible relationship between low levels of CoQ_10_ and ubiquinol in sarcopenia [122].

Despite the multiple reports on the effects of CoQ_10_ supplementation on aging-related oxidative markers, reduction in biomarkers related to inflammation and in DNA repair mechanisms reports there is a great need for more controlled studies in an older population to determine effectiveness of CoQ_10_ as an anti-aging therapy [97] and also to determine the tissue CoQ_10_ levels in the human species during senescence.

### 3.3. CoQ_10_ and Specific Conditions Associated with Age

The case for beneficial effects of CoQ_10_ or ubiquinol supplementation is stronger for a number of aging-related diseases many of which have documented mitochondrial dysfunction or CoQ_10_ deficiency. Despite this, the majority of studies proving therapeutic effects of CoQ_10_ supplementation were carried out in animal models [25].

Early studies were carried out with CoQ_10_ although often information on the formulation used is lacking (it is known that ubiquinol can be 3 to 4 times better absorbed than ubiquinone). Also, the doses used in most of the studies were lower than the doses studied for primary CoQ_10_ deficiency (up to 1200 mg/day) and in secondary CoQ_10_ deficiency studies. The formulation and absorption will affect studies of treatment efficacy. A colloidal-Q10 formulation has been shown to have a better enteral absorption and bioavailability in human tissues, and also the vehicle of the active ingredient can impact the results of trials [123].

#### 3.3.1. Neurodegenerative Disorders

Neurodegenerative diseases involve neuro-inflammation and oxidative stress, with ROS accumulation and mitochondrial dysfunction [83,124,125].

The data supporting mitochondrial cofactor supplementation in aging and the aging-related diseases, including Alzheimer disease, Parkinson disease, amyotrophic lateral sclerosis, and multiple sclerosis, are addressed in focused review articles in this supplement. A systematic review published in 2014 reviewed 16 articles and concluded that there is no available data from randomized controlled trials (RCTs) to support the use of mitochondrial supplements for Parkinson disease, atypical Parkinsonism, Huntington disease, and Friedreich ataxia [126].

Previous studies have shown that patients with Lewy’s body disease and amyotrophic lateral sclerosis have lower CoQ_10_ plasma levels and Alzheimer disease patients have reduced levels of CoQ_10_ in the cerebral spinal fluid [127,128].

In Huntington disease, increased levels of cortical lactate were observed, which were normalized after 2 months of Coenzyme Q_10_ oral supplementation at a dose of 360 mg/day. In another publication, after discontinuation of the supplement, the lactate levels rose again [67,124]. This study does provide evidence for CNS penetration of administered CoQ_10_ in humans. These and other data lead to a multicenter RCT with 609 patients, which could not identify a benefit in Functional Capacity Score and time to death after 60 months of 2400 mg per day of CoQ_10_ supplementation compared to placebo [129]. Thus, currently, there is insufficient evidence to support CoQ_10_ supplementation as a treatment to delay neurodegeneration in Huntington disease even in the early stages of the disease.

A similar situation occurred in Parkinson disease. An early study found that patients had decreased levels of α-tocopherol and CoQ_10_ in plasma and cerebrospinal fluid with increased levels of lipoprotein oxidation compared to controls in a 2004 study of 161 subjects. [130]. Mitochondrial complexes I and II/III were reduced in platelet mitochondria from early Parkinson disease patients [131]. A more recent study confirmed the association of Parkinson’s disease and CoQ_10_ deficiency using Functional Intracellular Assay, when compared to matched controls for age and gender [132]. A phase 2 study in 80 subjects at early or mid-stage Parkinson disease showed a beneficial effect from supplementation with CoQ_10_ in progressive doses of 300, 600, 1200 mg/day for 16 months. There was an improvement in the ADL UPDRS (Activity Of Daily Living Unified Parkinson Disease Rating Scale) and Schwab and England scales compared to placebo (+11.99), with highest dose (+6.69) having the best effect [133]. This lead to a phase III randomized, placebo-controlled, double-blind clinical trial at 67 North American sites by the Parkinson Study Group which used doses up to 2400 mg/day of CoQ_10_ vitamin E in the dose of 1200 IU/d (to enhance the absorption of the lipophilic coenzyme) however this large study did not find convincing evidence to support CoQ_10_ treatment for this disease [7]. A systematic review and meta-analysis from 2016 could not find sufficient evidence in five selected RCTs to support the use of ubiquinone (300 mg/day to 2400 mg/day) to decelerate the progression of Parkinson’s disease or improve symptoms [134].

In aged, cognitively impaired mice, CoQ_10_ supplementation does improve special learning and attenuates oxidative damage [135]. In Alzheimer Disease, the Alzheimer’s Disease Cooperative Study could not identify benefits in the levels of oxidative stress and neurodegeneration biomarkers in CSF (cerebrospinal fluid) with a dose of 400 mg of CoQ_10_ 3 times/day for 16 weeks for patients [136]. To date, chronic large-scale trials of CoQ_10_ have not been carried out in Alzheimer disease.

#### 3.3.2. Cardiovascular Disease 

A meta-analysis reviewed randomized controlled trials in healthy adults; two trials reported reduction in systolic blood pressure, and no evidence for reduction in diastolic blood pressure was reported. Another trial included in this meta-analysis failed to show an effect of CoQ_10_ supplementation on the lipid profile (LDL: low-density lipoprotein cholesterol, HDL: high-density lipoprotein cholesterol, and triglycerides) [137]. Another meta-analysis from 2016 concluded that there is not enough evidence to support CoQ_10_ use to treat hypertension [138]. For dyslipidemia, no relationship was found between CoQ levels in hyperlipidemic and normolipidemic, older women and no association with body mass index was found [139]. Statins are well known to reduce CoQ_10_ levels because of inhibition of 3-hydroxy-3-methylglutaryl-coenzyme A (HMG-CoA) reductase. This enzyme is the first step in the mevalonic acid synthesis pathway, which is necessary for the synthesis of cholesterol but also the isoprenoid side-chain of CoQ_10_ [140].

Patients with mitochondrial myopathy do not tolerate statin treatment, often developing exercise intolerance and myalgia when treated with statins. These symptoms may improve with CoQ_10_ oral supplementation [141,142,143]. Statins were also found related to a reduction in complex III activity. However, the exact mechanism for the mitochondrial myotoxicity of statins is not well understood and other factors than CoQ_10_ can be involved, such as genetic polymorphisms [144].

There is evidence that cardiac function in the elderly may be improved by CoQ_10_ treatment. This effect may be related to the documented fall in cardiac CoQ_10_ levels in the aged heart [114]. A meta-analysis reviewed eight clinical trials and concluded that patients submitted to cardiopulmonary bypass have a decreased requirement for ionotropic drugs and a reduced chance of ventricular arrhythmia if receiving CoQ_10_ supplementation [145].

The best case for CoQ_10_ supplementation is in cardiovascular disease. It was recently reported that 12 years after a 4-year double-blind treatment protocol in the elderly co-supplemented with selenium (200 µg) and CoQ_10_ (200 mg/day) (ubiquinone in a softgel vehicle of vegetable oils) the treated subjects still had a reduced cardiovascular mortality (38.7% in placebo-treated group versus 28.1% in CoQ-treated group) [146]. The Q-SYMBIO trial enrolled 420 patients and showed that CoQ_10_ 100 mg 3 times daily for 2 years plus standard therapy reduced the risk of cardiovascular events, in this study measured as cardiovascular mortality (16% in placebo and 9% in the treated group) and hospitalization for heart failure compared to placebo, and also a positive change in the New York Heart Association (NYHA) functional classification (26% in placebo and 15% in the treated group) [147]. A recent review of the use of CoQ_10_ in heart failure is recommended [39].

#### 3.3.3. Endothelial Dysfunction

Endothelial function may be a risk factor for coronary artery disease and atherosclerosis. CoQ_10_ together with a Mediterranean diet was shown to improve markers of endothelial function in elderly patients [148,149].

#### 3.3.4. Renal Disease

Although ubiquinol can improve the endothelial dysfunction associated with the diabetic kidney disease (systolic blood pressure and urinary albumin) [150], and a trial with CoQ_10_ supplementation (1200 mg/day in dialysis patients) identified a reduction in a plasma indicator of oxidative stress (F2-isoprostane) [151], a meta-analysis failed to prove CoQ_10_ efficacy in avoiding the progression of diabetic kidney disease [152].

#### 3.3.5. Inflammation

Many aging-related diseases share a common physiologic pathway of chronic inflammation leading to oxidative stress, such as cardiovascular diseases, diabetes, cancer, and chronic kidney disease.

A recent meta-analysis reports a reduction in the plasma inflammatory biomarkers, C-reactive protein, IL-6, and TNF-α, after CoQ_10_ supplementation (60 to 500 mg/day, formulations described as CoQ10 or ubiquinol), for 1 week to 4 months in different inflammatory disorders (cardio and cerebral vascular disease, multiple sclerosis, obesity, renal failure, rheumatoid arthritis, diabetes, and fatty liver disease). The same review reports that CoQ_10_ decreases other biomarkers for inflammation and inflammatory cytokines [30]. Although CoQ_10_ treatment has been shown to improve markers of inflammation, a benefit of chronic treatment for the diseases associated with inflammation has not been demonstrated.

CoQ_10_ (100 mg/day) supplementation for 2 months decreased the levels of TFN-α in rheumatoid arthritis patients compared to placebo, [153]. A more recent systematic review and meta-analysis reported CoQ_10_ supplementation between 60 to 300 mg/day (no extra information about formulations) was associated with a slightly drop in C-reactive protein levels and a significant decrease in in IL-6 levels [154].

Down syndrome patients have an abnormal pro-inflammatory profile (increased IL-6 and TNF-α) [36,37] and reduced CoQ_10_ levels, and supplementation reduced markers of oxidative stress and mitochondrial dysfunction [38,155].

#### 3.3.6. Osteoporosis 

Animal and human studies have demonstrated that benefits of CoQ_10_ supplementation have a beneficial profile for osteoporosis [156,157,158].

#### 3.3.7. Cancer

There is evidence of the relationship between some cancers and reduced CoQ_10_ levels in blood, particularly breast cancer [159], myeloma [160], melanoma [161], and follicular and papillary thyroid carcinomas [162]. There is also evidence that CoQ_10_ supplementation modulates phospholipid hydroperoxide glutathione peroxidase gene expression, free radical production, and can decelerate the growth of tumor cells in a prostate cancer line (PC3 line) [163].

## 4. Conclusions

There is still much to be learned about the pathophysiology of the process of aging. There are well documented reductions of tissue CoQ_10_ in senescence. It is not known if low CoQ_10_ is an effect of aging, perhaps matching the fall in mitochondrial electron transport function or a contributing cause to the aging process. There is accumulating evidence that some diseases of aging may benefit from supplemental ubiquinol or CoQ_10_ treatment. Studies to date have supported the safety and the potential of CoQ_10_ in reducing oxidative stress biomarkers. There remains a lack of adequate large-scale clinical trials preferably utilizing ubiquinol as the better absorbed form of CoQ_10_. Despite the lack of evidence, large numbers of people in the population are taking CoQ_10_ and other vitamins and cofactors in the hope that these agents will slow senescence and expand longevity.
